# Effect of contact resistance on the electrical conductivity of polymer graphene nanocomposites to optimize the biosensors detecting breast cancer cells

**DOI:** 10.1038/s41598-022-09398-0

**Published:** 2022-03-30

**Authors:** Yasser Zare, Kyong Yop Rhee

**Affiliations:** 1 Biomaterials and Tissue Engineering Research Group, Department of Interdisciplinary Technologies, Breast Cancer Research Center, Motamed Cancer Institute, ACECR, Tehran, Iran; 2 Department of Mechanical Engineering (BK21 Four), College of Engineering, Kyung Hee University, Yongin, Republic of Korea

**Keywords:** Nanoscience and technology, Graphene, Nanoscale materials

## Abstract

This study focuses on the contact regions among neighboring nanoparticles in polymer graphene nanocomposites by the extension of nanosheets. The resistance of graphene and the contact zones represent the total resistance of the prolonged nanosheets. Furthermore, the graphene size, interphase depth, and tunneling distance express the effective volume portion of graphene, while the onset of percolation affects the fraction of percolated nanosheets. Finally, a model is developed to investigate the conductivity of the samples using the graphene size, interphase depth, and tunneling size. In addition to the roles played by certain factors in conductivity, the experimental conductivity data for several samples confirm the conductivity predictions. Generally, the polymer sheet in tunnels determines the total resistance of the extended nanosheets because graphene ordinarily exhibits negligible resistance. In addition, a large tunnel positively accelerates the onset of percolation, but increases the tunneling resistance and attenuates the conductivity of the nanocomposite. Further, a thicker interphase and lower percolation threshold promote the conductivity of the system. The developed model can be applied to optimize the biosensors detecting the breast cancer cells.

## Introduction

Graphene has the ability to combine the unusual electrical conductivity of carbon nanotubes (CNT) and excellent barrier properties of layered clays with significant mechanical stiffness^1–12^, allowing for the production of high-quality polymer nanocomposites for advanced multi-functional applications^13–22^. However, the dispersion of graphene and the formation of conductive networks in thermoplastic polymer matrices are easier than those of rubbers, owing to the complexity of network creation in cross-linked rubber matrices. Certain studies have shown that graphene nanocomposites exhibit a relatively low percolation threshold and more optimized electrical conductivity than CNT^23^. Generally, the high surface energy of graphene and their strong interactions attenuate their uniform dispersal in the polymer medium^24–28^.

The conductivity of nanocomposites is commonly governed by the concentration, dimensions, conductivity, and dispersion features of nanoparticles^29,30^. Many models have been developed to assume the effects of various variables, such as polymer-filler interfacial energy (affecting dispersion level), tunneling effect, agglomeration, and waviness on the conductivity of polymer nanocomposites^31–37^. These models can offer guidelines for determining effective parameters on the conductivity of nanocomposites. Numerous studies have used the conventional power-law model to predict the percolation onset and an exponent using the tested conductivity for graphene-based nanocomposites^38–40^; however, this model disregards innovative features such as tunnels and interphase districts.

The tunneling effect governs the electrical conductivity of graphene-filled nanocomposites because electrons can be transported between adjacent nanosheets through a tunneling mechanism, even when they are not physically joined^41–44^. This implies that even a short distance between nanoparticles can form conductive paths to promote conductivity. Accordingly, the onset of percolation in the nanocomposites does not depend solely on the dimensions of nanoparticles, because it can be changed by the tunneling distance. The filler dimensions, contact area, tunneling distance, and polymer matrix affect the contact resistance^45^. Furthermore, the tunneling resistance was found to increase rapidly as the thickness of insulating layer between the two nanoparticles increases^46^.

The large surface area of nanofillers frequently results in the development of interphase districts among the polymer media and nanofillers in nanocomposites^47–51^. The effects of the interphase depth and toughness on the potency of polymer nanocomposites have been studied previously^52–55^. Interestingly, the interphase district can create networks in the nanocomposite, which shifts the onset of percolation and enlarges the nets^56,57^. This necessitates the assumption of an interphase role at the onset of percolation and conductivity. However, to the best of our knowledge the limited number of models in this area cannot simulate the tunnels and interphase with respect to the conductivity of nanocomposites.

Biosensors with high sensitivity and good selectivity are important in the medical fields such as cancer. Recently, various nanomaterials were used to design the effective biosensors for the detection of breast cancer cells^58,59^. Graphene-based nanocomposites are ideal for fabricating the low-cost and efficient electrodes, because of their unique properties causing high sensitivity, good selectivity and low-limit detection^60–62^. However, there is not a simple model predicting the electrical conductivity of graphene-filled nanocomposites in biosensors.

In this study, the contact region between adjacent nanoparticles is considered by increasing the diameter of graphene nanosheets. The resistances of the contact region and graphene are assumed in a simple model to estimate conductivity. Moreover, the filler dimensions express the percolation threshold and effective filler concentration. Therefore, the developed model can predict conductivity using numerous graphene, interphase, and tunnel factors. Empirical records and parametric analyses are used to examine the suggested model. The developed model can provide precise prediction of conductivity, suggesting valuable strategies for the optimization of multifunctional products.

## Development of equations

The contact regions between the nanoparticles can be considered by extending the nanosheets. The total resistance of the extended nanosheets is calculated and its effects on the conductivity of nanocomposites are examined.

**Figure 1:**
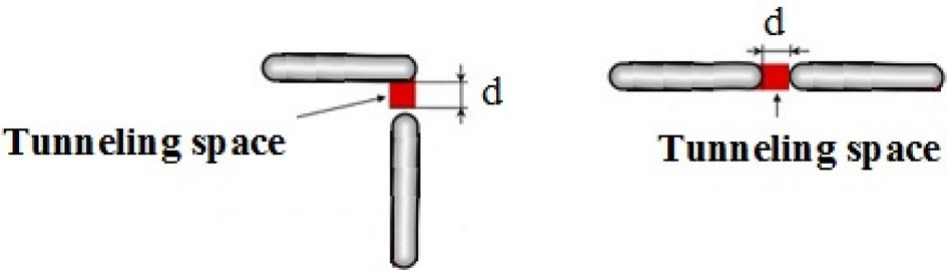
Two types of contact spaces in polymer graphene nanocomposite.

The two possible configurations of contacts between randomly dispersed nanosheets are expressed as crossing and overlapping, as shown in Fig. 1. The contact surface areas in the two cases are equal to the cross-sectional area of the nanosheet.

The total resistance of an extended nanosheet is suggested to be:

1 $$ R = R_{f} + R_{c} $$

where R_f_ and R_c_ represent graphene and contact resistance, respectively,

R_f_ is expressed as^63^:

2 $$ R_{f} = \frac{D}{{S\sigma_{f} }} = \frac{1}{{t\sigma_{f} }} $$

where D, S, σ_f_, and t are the diameter, cross-sectional area, conductivity and thickness of the graphene nanosheet, respectively.

In addition, the contact resistance includes the intrinsic resistance of the nanosheets on both sides of the tunneling spaces and the tunneling resistance introduced by the insulating matrix layer into the tunneling regions. Therefore, the contact resistance is expressed by the resistances of the graphene fraction between two contacts (R_g_) and the polymer tunneling resistance (R_t_) as:

3 $$ R_{c} = R_{g} + R_{t} $$

R_g_ was expressed as^45^:

4 $$ R_{g} = \frac{1}{{2t\varphi_{f} \sigma_{f} }} $$

where $\varphi_{f}$ denotes the volume portion of the filler.

The tunneling resistance also depends on the thickness and surface area of the insulating layer as:

5 $$ R_{t} = \frac{\rho d}{S} = \frac{\rho d}{{tD}} $$

where ρ shows the tunneling resistivity and d is the tunneling distance. The polarity of polymers has tremendous effect on the electron transfer, thereby controlling the ρ as tunneling resistivity.

Accordingly, the contact resistance is expressed as:

6 $$ R_{c} = \frac{1}{{2t\varphi_{f} \sigma_{f} }} + \frac{\rho d}{{tD}} $$

which represents the total resistance of the extended nanosheets (Eq. 1) as:

7 $$ R = \frac{1}{{t\sigma_{f} }} + \frac{1}{{2t\varphi_{f} \sigma_{f} }} + \frac{\rho d}{{tD}} $$

The conductivity of an extended nanosheet is also expressed by restructuring Eq. 2 as follows:

8 $$ \sigma_{ext} = \frac{D + 2d}{{tDR}} = \frac{D + 2d}{{tD\left(\frac{1}{{t\sigma_{f} }} + \frac{1}{{2t\varphi_{f} \sigma_{f} }} + \frac{\rho d}{{tD}}\right)}} $$

Because D >> d and 1/σ_f_ is negligible, σ_ext_ can be expressed as follows:

9 $$ \sigma_{ext} = \frac{1}{{\frac{1}{{2\varphi_{f} \sigma_{f} }} + \frac{\rho d}{D}}} $$

The conductivity of the extended nanosheets assuming the graphene and contact regions are used to predict the conductivity using a simple model.

An equation for nanocomposite conductivity due to accidental dispersal of CNTs^32^ is proposed as:

10 $$ \sigma = \sigma_{0} + \frac{{f\varphi_{f} \sigma_{f} }}{3} $$

where σ_0_ is polymer matrix conductivity and f is the portion of particles in the nets. The σ_0_ (10^−13^–10^−16^ S/m) can be disregarded when it is too low. In addition, Eq. 10 can be used for graphene-filled systems.

When the total conductivity of the extended nanosheets from Eq. 9 is considered in the latter model, the conductivity is expressed as:

11 $$ \sigma = \frac{{f\varphi_{f} }}{{3\left(\frac{1}{{2\varphi_{f} \sigma_{f} }} + \frac{\rho d}{D}\right)}} $$

indicating the correlation of the conductivity to graphene, network and tunneling properties.

As mentioned, the interphase regions around graphene can promote the effects of nanoparticles on conductivity by network contribution.

The interphase volume fraction in nanocomposites^64^ is calculated as:

12 $$ \varphi_{i} = \varphi_{f} \left(\frac{{2t_{i} }}{t}\right) $$

where t_i_ is the interphase depth.

The optimal graphene volume portion in the samples contains filler and interphase concentrations^63^ because both graphene and the surrounding interphase control the conductivity of nanocomposites.

13 $$ \varphi_{eff} = \varphi_{f} + \varphi_{i} = \varphi_{f} \left(1 + \frac{{2t_{i} }}{t}\right) $$

Additionally, the onset of percolation in randomly distributed graphite nanosheets in polymer nanocomposites has been proposed^65^ as:

14 $$ \varphi_{p} = \frac{{27\pi D^{2} t}}{{4(D + d)^{3} }} \cong \frac{27\pi t}{{4D}} $$

This equation does not reflect the interphase and tunneling roles in the percolation phenomenon, while they form around the nanosheets and shift the onset of percolation.

The subsequent equation can be developed based on interphase and contact districts as:

15 $$ \varphi_{p} = \frac{27\pi t}{{4D + 2(Dt_{i} + Dd)}} $$

expressing the effects of the filler size, interphase depth, and tunneling size on the percolation value. This equation is applied to estimate the interphase and tunneling dimensions of the examples.

Furthermore, only a portion of the nanoparticles was employed in the nets at the onset of percolation, whereas others were detached in the sample. f, the portion of net in the sample^66^, was expressed as:

16 $$ f = \frac{{\varphi_{f}^{1/3} - \varphi_{p}^{1/3} }}{{1 - \varphi_{p}^{1/3} }} $$

f is determined assuming interphase and contact zones in effective filler fraction and the onset of percolation (Eqs. 13 and 15) as:

17 $$ f = \frac{{\varphi_{eff}^{1/3} - \varphi_{p}^{1/3} }}{{1 - \varphi_{p}^{1/3} }} $$

Finally, the model developed using Eq. 11 can be presented by reflecting the interphase and tunneling impacts as:

18 $$ \sigma = \frac{{f\varphi_{eff} }}{{3\left(\frac{1}{{2\varphi_{eff} \sigma_{f} }} + \frac{\rho d}{D}\right)}} $$

which expresses a complete model for the conductivity of graphene-filled systems according to the specifications of the graphene, interphase, and tunnels.

## Results and discussion

### Experimented and predicated results of conductivity

The measured conductivities of several nanocomposites from previous studies are used to evaluate the developed equations. Four graphene specimens containing poly(vinyl alcohol) (PVA) (D = 2 μm, t = 2 nm and $\varphi_{p}$ = 0.0035, chemically reduced graphene oxide)^67^, polyimide (PI) (D = 5 μm, t = 3 nm and $\varphi_{p}$ = 0.0015, reduced graphene oxide)^68^, acrylonitrile–butadiene–styrene (ABS) (D = 4 μm, t = 1 nm and $\varphi_{p}$ = 0.0013, chemically reduced graphene oxide)^29^ and poly(ethylene terephthalate) (PET) (D = 2 μm, t = 2 nm and $\varphi_{p}$ = 0.005, thermally reduced graphene oxide)^69^ are considered. The onset of percolation ($\varphi_{p}$) was obtained as the volume fraction of graphene where the conductivity sharply increased. By applying the filler dimensions to Eq. 15, the values of the interphase thickness and tunneling distance are predicted. The values of (t_i_, d) for the PVA, PI, ABS, and PET samples are (5, 5), (7, 9), (3, 3), and (3, 4) nm, respectively. PI/graphene nanocomposite yields the highest values of (t_i_, d), whereas ABS/graphene yields the smallest values. Therefore, it is possible to estimate and evaluate the extents of the interphase and tunneling based on the onset of percolation. Generally, a low percolation threshold is attained in samples comprising narrow and large nanosheets, profuse interphase, and large tunnels (Eq. 15), as calculated for the reported samples. However, disregarding the interphase and tunnels inaccurately predicts the percolation threshold. Accordingly, the interphase and tunnels mainly change the percolation beginning of graphene, thereby affecting conductivity.

**Figure 2:**
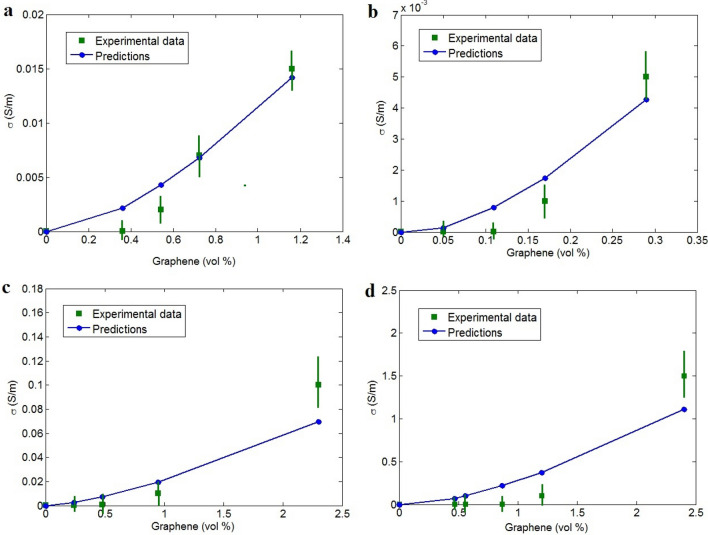
Application of the developed model to estimate the conductivity of (**a**) PVA^67^, (**b**) PI^68^, (**c**) ABS^29^ and (**d**) PET^69^ graphene systems.

The values of (t_i_ and d) can be used to calculate the effective filler concentration (Eq. 13) and f as the fraction of networked nanosheets (Eq. 17). Consequently, the conductivities of the aforementioned samples is calculated using the developed model at σ_f_ = 10^5^ S/m. Figure 2 shows the experimental (from references) and theoretical conductivity values for these examples. Adequate agreement between the experimental results and predictions confirm the accuracy of the model. Consequently, the developed model originating from the resistances of the contact regions between adjacent nanosheets can be used for the assessment of conductivity in polymer–graphene nanocomposites. The values of ρ are calculated to be 200, 100, 500 and 5 Ω m for PVA, PI, ABS and PET graphene nanocomposites, respectively. Therefore, the highest and lowest tunneling resistivity is observed in the ABS/graphene sample and PET/graphene, respectively. According to Fig. 2, the maximum conductivity is observed in PET/graphene sample, whereas ABS/graphene exhibits low conductivity at high graphene concentrations. The high tunneling resistivity decreases electron movement via tunneling spaces, which attenuates the conductivity of the nanocomposites. The data on the tunneling and interphase characteristics are significant and reasonable, supporting the validity of the developed model.

### Justifications for the impact of parameters on the conductivity

The significance of all the factors on conductivity is described to validate the developed model. The average values of the variables in all plots are t = 2 nm, $\varphi_{f}$ = 0.01, D = 2 μm, t_i_ = 4 nm, σ_f_ = 10^5^ S/m, d = 5 nm, and ρ = 200 Ω m.

**Figure 3:**
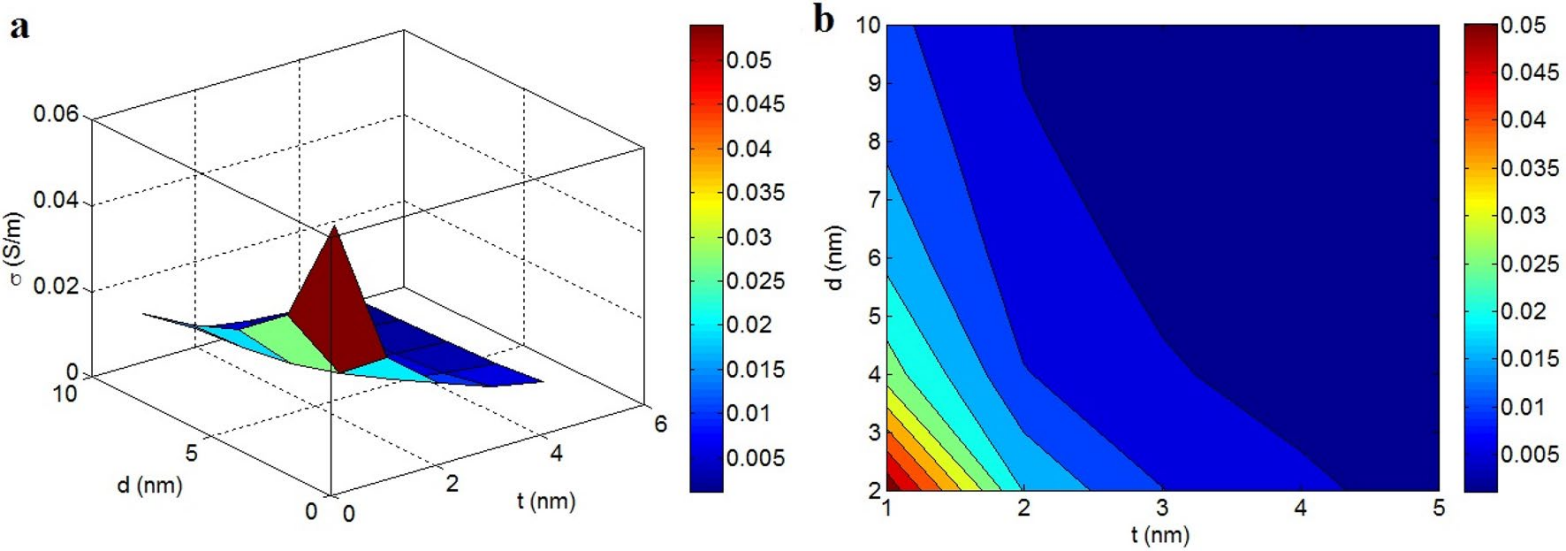
Conductivity by the variation of t and d provided by the new model: (**a**) 3D and (**b**) contour patterns.

Figure 3 portrays the roles of “t” and “d” in the conductivity according to the developed model. A deprived conductivity is detected at the high ranks of t and d, while the highest conductivity is suggested at the lowest ranges of these factors. t > 2.5 nm and d > 6 nm meaningfully lower the conductivity to 0.001 S/m. Nonetheless, the maximum conductivity as 0.05 S/m is acquired at t = 1 nm and d = 2 nm. As a result, thin nanosheets and a small tunneling distance can produce high conductivity in the nanocomposite.

Thin nanosheets favorably govern the conductivity of the nanocomposite because they yield high operational filler attentiveness (Eq. 13) and low percolation threshold (Eq. 15), which ultimately yield the desirable f (Eq. 17). In other words, thin graphene accelerates the percolation and enlarges the interphase areas, thereby resulting in the formation of highly conductive nets in the nanocomposite. Therefore, reedy nanosheets increase conductivity through the formation of efficient graphene networks. In addition, despite lowering the percolation threshold (Eq. 15), a large tunnel significantly increases the tunneling resistance caused by the insulated polymer matrix (Eq. 5). In fact, a large tunnel attenuates the transportation of electrons between adjacent nanosheets because the tunnels contain an insulated polymer. The inverse relationship between the conductivity and tunneling size has also been reported in other studies^70,71^, and a few studies have shown a direct relationship between these factors^65^. However, the large tunneling spaces limit the transfer of charges because of the intrinsic insulating nature of the polymer matrix in these regions. Therefore, the developed equation rationally expresses the impacts of “t” and “d” on the conductivity.

**Figure 4:**
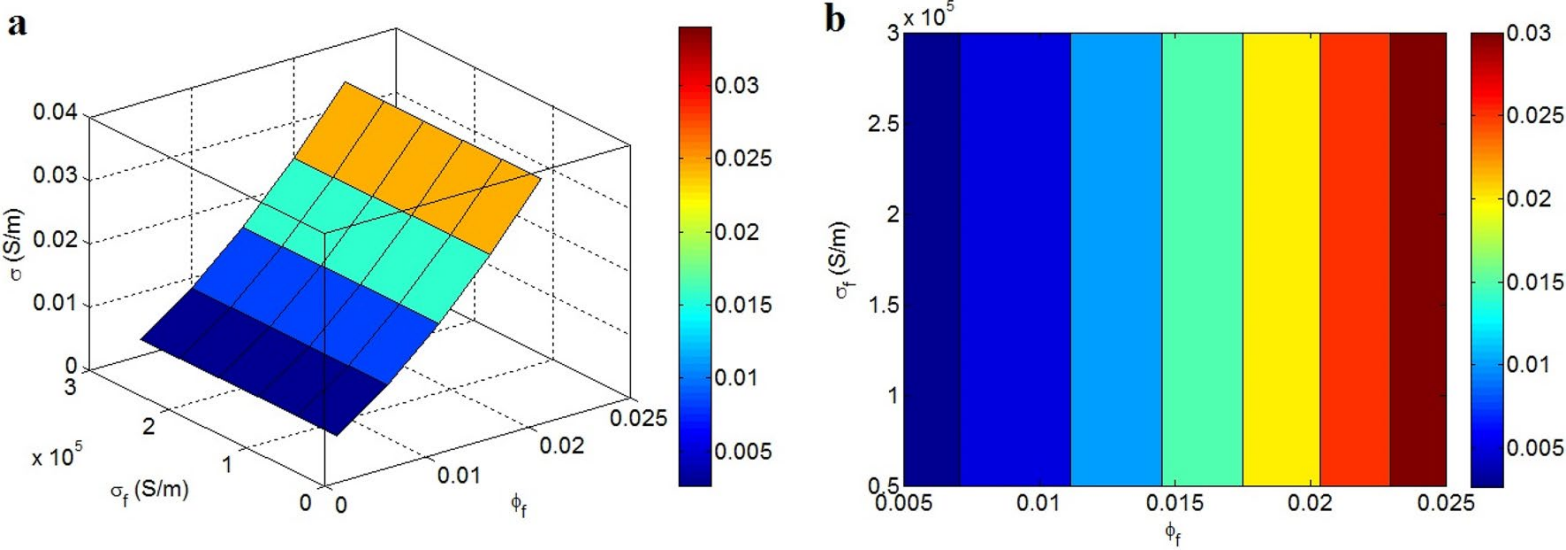
Disparities of conductivity at dissimilar arrays of $\varphi_{f}$ and σ_f_: (**a**) 3D and (**b**) contour strategies.

The variations in conductivity by $\varphi_{f}$ and σ_f_ are also shown in Fig. 4. The conductivity only depends on $\varphi_{f}$ and is not affected by σ_f_. $\varphi_{f}$ > 0.023 produces the conductivity of 0.03 S/m, whereas low $\varphi_{f}$ < 0.007 results in σ = 0.003 S/m. So, high filler attentiveness directly improves conductivity, but cannot be altered by the conductivity of graphene.

An extraordinary concentration of nanoparticles logically increases conductivity because it increases the segment of the conductive phase in the sample, which produces conductive paths for electron transfer. In reality, the concentration of conductive graphene in the nanocomposite is significant because only graphene controls the conductivity of the nanocomposite in the presence of an insulated medium. However, a significantly higher concentration of nanoparticles after percolation beginning negligibly affects conductivity because they fail to considerably change the performance of networks^34^. Conversely, the significant conductivity of graphene nanosheets compared to polymer matrices mainly decreases the resistance of the graphene nanosheets in the networks and contacts. Accordingly, the high conductivity of graphene considerably minimizes the R_f_ (Eq. 2) and R_g_ (Eq. 4) in Eq. 7; therefore, σ_f_ cannot manipulate the conductivity of nanocomposite. However, certain studies have reported that the filler conductivity controls the conductivity of the sample because they commonly do not pay attention to the contact resistance^29,67,68^. Consequently, the developed model correctly expresses the roles of $\varphi_{f}$ and σ in the conductivity of polymer graphene nanocomposites.

**Figure 5:**
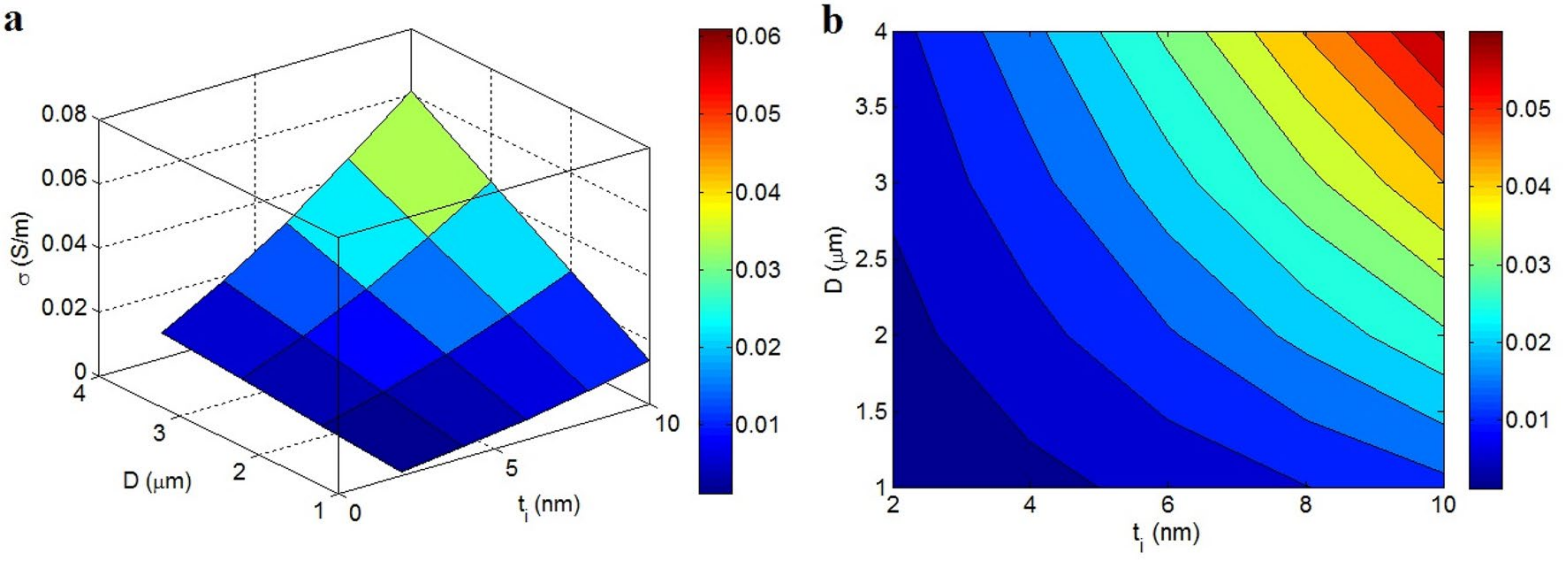
Conductivity of nanocomposites by “t_i_” and “D”: (**a**) 3D and (**b**) contour diagrams.

Figure 5 portrays the reliance of conductivity on t_i_ and D. The small ranks of these factors decrease conductivity, but extraordinary conductivity is observed at high ranges of these terms. t_i_ < 3 nm and D < 1.75 μm produce an insulated nanocomposite, but t_i_ = 10 nm and D = 4 μm cause the conductivity of 0.06 S/m. Accordingly, both t_i_ and D as interphase thickness and graphene diameter directly alter conductivity. This evidence is reasonable because these parameters positively affect the properties of conductive networks.

A thick interphase enhances the effective filler concentration (Eq. 13), and decreases the percolation level (Eq. 15) because the interphase can create and enlarge the nets in the nanocomposite along with the nanoparticles. In other words, large interphase regions can enhance the fraction of percolated nanosheets (Eq. 17), and plays a major role in the conductivity of the nanocomposite. However, the thin interphase around the nanoparticles insignificantly affects the efficiency of the nets and the conductivity of the samples. The effects of interphase depth on the percolation beginning and rigidity of CNT-filled nanocomposites have been reported in previous studies^72^, but this interesting item has not been studied for polymer graphene samples.

The direct character of D in the conductivity is justifiable because large nanosheets lower the percolation threshold, which changes the size and density of filler networks. Large nanosheets can produce bulky nets in the sample, which facilitates electron transportation. In addition, the large nanosheets diminish the tunneling resistance introduced by the polymer matrix (Eq. 5) because they produce a large contact area in the tunneling spaces. In fact, large graphene nanosheets decrease the negative effect of tunneling resistance on conductivity. Therefore, the novel model suitably displays the effect of D on conductivity. The optimistic effect of the graphene diameter on the percolation threshold was reported in certain studies^65,73^, but its direct role in the conductivity of nanocomposites has been limitedly discussed in previously studies.

**Figure 6:**
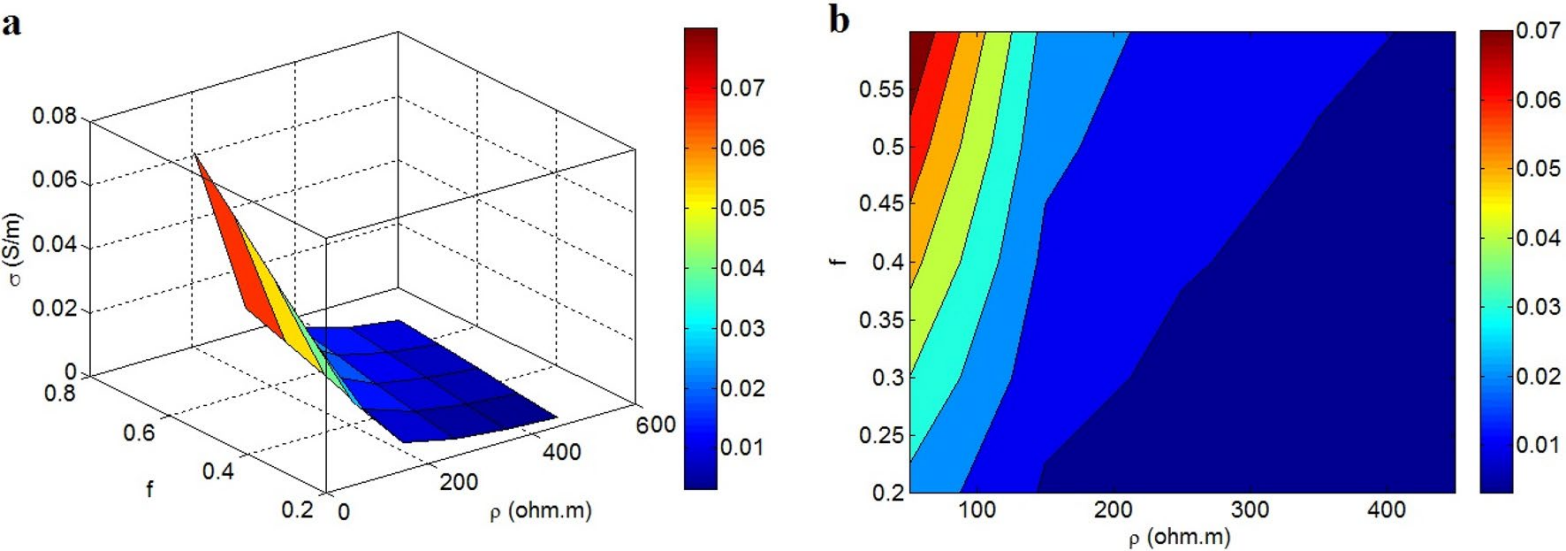
Effects of ρ and f on conductivity by (**a**) 3D and (**b**) contour illustrations.

Figure 6 also displays the changes in conductivity due to ρ and f. The optimal conductivity is obtained by the slightest ρ and the highest f because ρ = 50 Ω · m and f = 0.6 lead to a conductivity of 0.07 S/m. However, high ρ and low f reduce the conductivity. Consequently, the polymer tunneling resistivity and fraction of percolated nanoparticles inversely and directly influence the conductivity of nanocomposite, respectively.

A high ρ enhances the general resistance of the extended graphene in the nanocomposite based on Eq. 5. In addition, a high level of σ_f_ decreases the resistances of other parts in R, as mentioned. Therefore, ρ significantly changes the conductivity and resistance of extended graphene, which play a key role in the conductivity of the entire nanocomposite. Clearly, a high ρ indicates high resistance against the transfer of electrons in the tunneling spaces, which decreases the conductivity of nanocomposite. Moreover, f reasonably governs conductivity because it represents the scope and density of the conductive nets in the sample. Meanwhile, the extent and efficiency of conductive networks determine the level of charge transportation and electrical conductivity in nanocomposites^74,75^; therefore, more optimized conductivity owing to relatively high f is logical. So, the present model accurately demonstrates the associations of conductivity to ρ and f.

**Figure 7:**
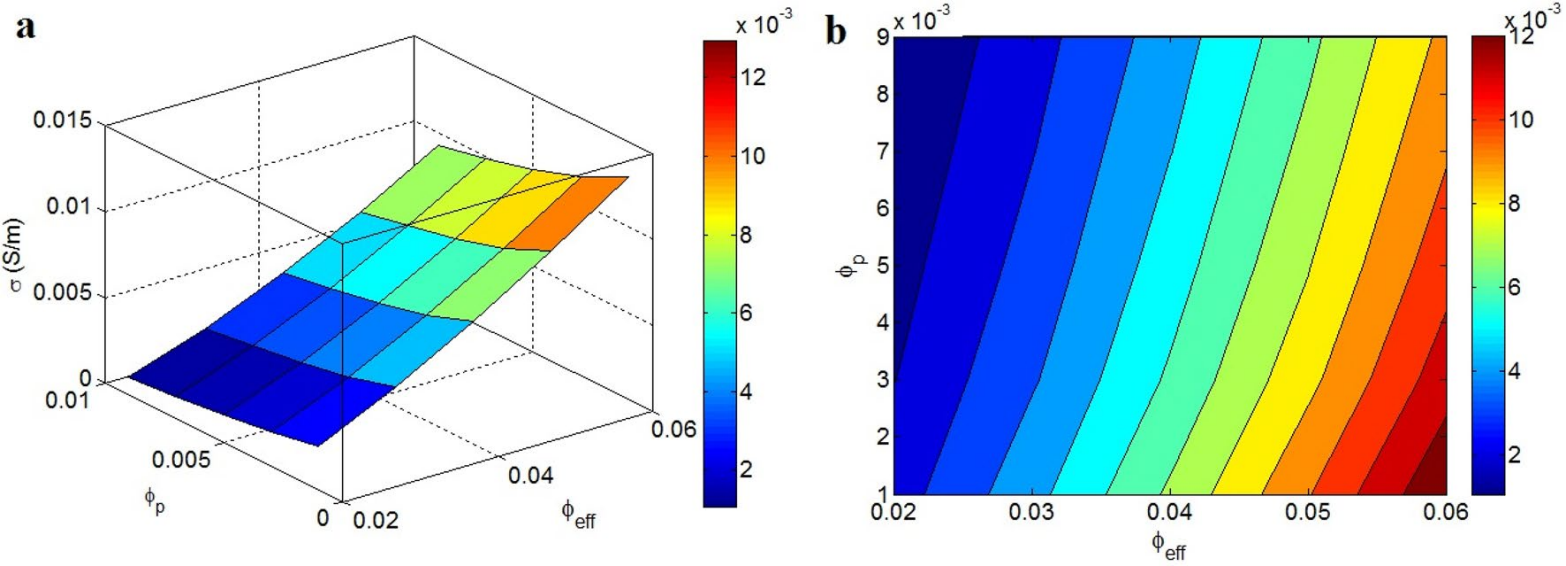
Correlations of conductivity to “$\varphi_{eff}$” and “$\varphi_{p}$”: (**a**) 3D and (**b**) contour schemes.

Finally, the conductivity is plotted at different values of $\varphi_{eff}$ and $\varphi_{p}$ in Fig. 7. Remarkable conductivity is detected at high $\varphi_{eff}$ and low $\varphi_{p}$, whereas the conductivity declines at low $\varphi_{eff}$ and high $\varphi_{p}$. A conductivity of 0.012 S/m is obtained at $\varphi_{eff}$ = 0.06 and $\varphi_{p}$ = 0.001, although the conductivity diminishes to 0.001 S/m at $\varphi_{eff}$ = 0.02 and $\varphi_{p}$ > 0.003. Accordingly, more effective filler faction and relatively low percolation beginning suggest a more desirable conductivity.

$\varphi_{eff}$ as the total portion of nanoparticles and interphase part determines the effectiveness of graphene in the nanocomposite. Moreover, a high $\varphi_{eff}$ causes an extraordinary f, indicating a large quantity of nanoparticles in the nets. Consequently, $\varphi_{eff}$ is a symbol of the level of interphase region regulating the magnitude of the conductive networks, which unquestionably governs conductivity. In addition, $\varphi_{p}$ is the essential fraction of nanosheets required to form the nets. Moreover, $\varphi_{p}$ diversely manages f, indicating that a low percolation threshold results in large nets. As a result, low percolation threshold positively influences the performance and efficiency of the graphene nets in the nanocomposite; therefore, a relatively high conductivity at a relatively low percolation level is reasonable. Therefore, the developed model justifiably determines the conductivity at the dissimilar $\varphi_{eff}$ and $\varphi_{p}$ ranges. The $\varphi_{eff}$ and $\varphi_{p}$ consistently rely on the filler and interphase extents. The graphene and interphase dimensions should be optimized by tuning the materials and processing factors to obtain high $\varphi_{eff}$ and low $\varphi_{p}$ values in the nanocomposite.

## Conclusions

The contact region between the nanosheets was assumed by extending the graphene nanosheets, and its effect on conductivity was evaluated. In addition, a simple model was developed to express the conductivity based on the graphene size, interphase depth, and tunneling distance. The experimental results from previous studies and parametric examinations were used to analyze the proposed model, confirming the approximations of the advanced model for the conductivity of the nanocomposites. The insulated polymer layer at the contact region mainly controls the resistance of the extended nanosheets and the conductivity of the nanocomposite because graphene intrinsically has a negligible resistance. Additionally, thinner and larger nanosheets and thicker interphases increase the conductivity of nanocomposites because they can enhance the effective filler fraction and decrease the percolation threshold to produce large nets. A high tunneling distance decreases the percolation beginning, but increases the tunneling resistance and diminishes electron transfer. Moreover, a relatively high effective filler portion and relatively low percolation threshold yield increased conductivity in nanocomposites. The advanced model is applicable to improve the performance of biosensors containing polymer graphene nanocomposites for detecting the breast cancer cells.
